# Supplementary material for: Mitochondrial Phylogenomics of Tenthredinidae (Hymenoptera: Tenthredinoidea) Supports the Monophyly of Megabelesesinae as a Subfamily
Source: Insects. 2021 May 26;12(6):495. doi: 10.3390/insects12060495 (PMC8227683; doi:10.3390/insects12060495)
Supplement: Supplementary file 1 [file insects-12-00495-s001.zip › insects-1186856-SI.pdf]

Supplementary materials

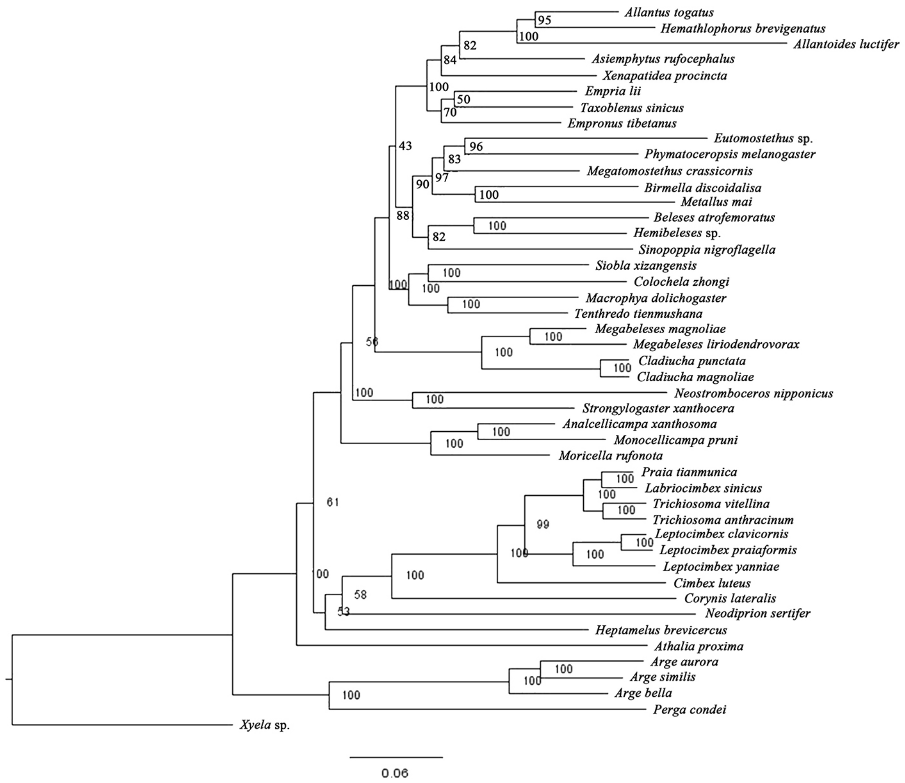

a

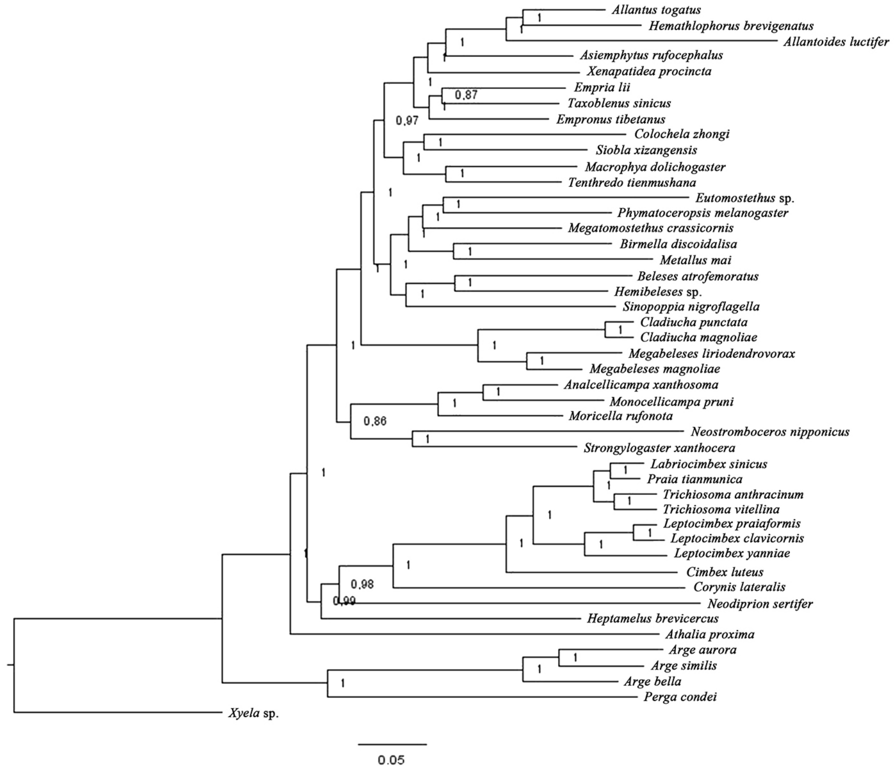

b

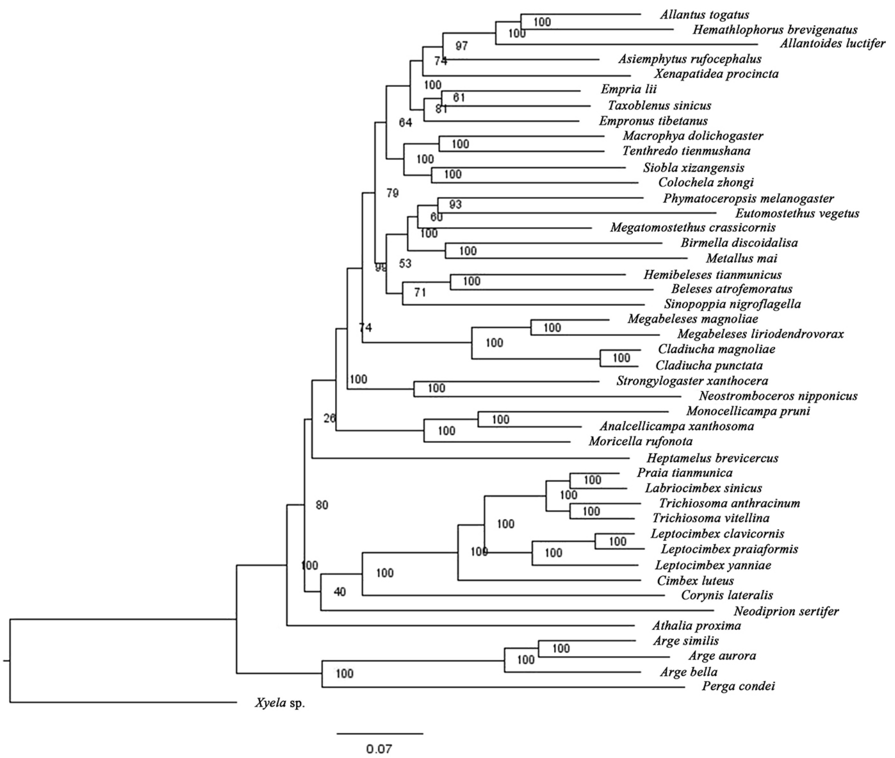
Allantus togatus
Hemathlophorus brevigenatus
Allantoides luctifer
Asiemphytus rufocephalus
Xenapatidea procincta
Empria lii
Taxoblenus sinicus
Empronus tibetanus
Macrophya dolichogaster
Tenthredo tienmushana
Siobla xizangensis
Colochela zhongi
Phymatoceropsis melanogaster
Eutomostethus vegetus
Megatomostethus crassicornis
Birmella discoidalisa
Metallus mai
Hemibeleses tianmunicus
Beleses atrofemoratus
Sinopoppia nigroflagella
Megabeleses magnoliae
Megabeleses liriodendrovorax
Cladiucha magnoliae
Cladiucha punctata
Strongylogaster xanthocera
Neostromboceros nipponicus
Monocellicampa pruni
Analcellicampa xanthosoma
Moricella rufonota
Heptamelus brevicercus
Praia tianmunica
Labriocimbex sinicus
Trichiosoma anthracinum
Trichiosoma vitellina
Leptocimbex clavicornis
Leptocimbex praiaformis
Leptocimbex yanniae
Cimbex luteus
Corynis lateralis
Neodiprion sertifer
Athalia proxima
Arge similis
Arge aurora
Arge bella
Perga condei
Xyela sp.
0.07

c

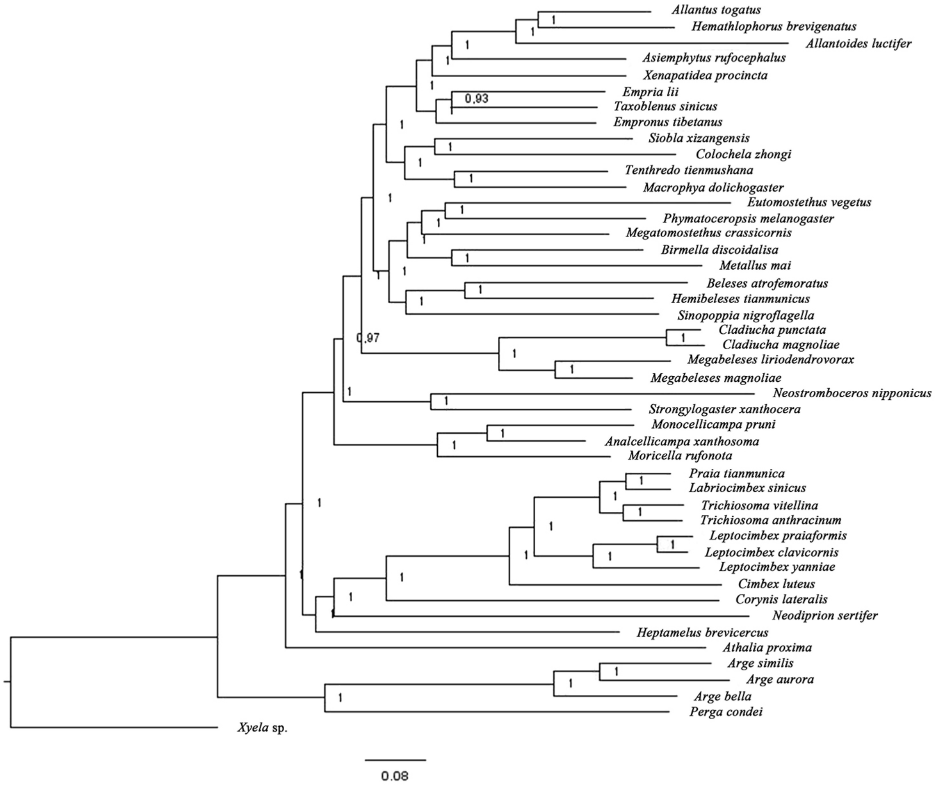
Allantus togatus
Hemathlophorus brevigenatus
Allantoides luctifer
Asiemphytus rufocephalus
Xenapatidea procincta
Empria lii
Taxoblenus sinicus
Empronus tibetanus
Siobla xizangensis
Colochela zhongi
Tenthredo tienmushana
Macrophya dolichogaster
Eutomostethus vegetus
Phymatoceropsis melanogaster
Megatomostethus crassicornis
Birmella discoidalisa
Metallus mai
Beleses atrofemoratus
Hemibeleses tianmunicus
Sinopoppia nigroflagella
Cladiucha punctata
Cladiucha magnoliae
Megabeleses liriodendrovorax
Megabeleses magnoliae
Neostromboceros nipponicus
Strongylogaster xanthocera
Monocellicampa pruni
Analcellicampa xanthosoma
Moricella rufonota
Praia tianmunica
Labriocimbex sinicus
Trichiosoma vitellina
Trichiosoma anthracinum
Leptocimbex praiaformis
Leptocimbex clavicornis
Leptocimbex yanniae
Cimbex luteus
Corynis lateralis
Neodiprion sertifer
Heptamelus brevicercus
Athalia proxima
Arge similis
Arge aurora
Arge bella
Perga condei
Xyela sp.
0.08

d

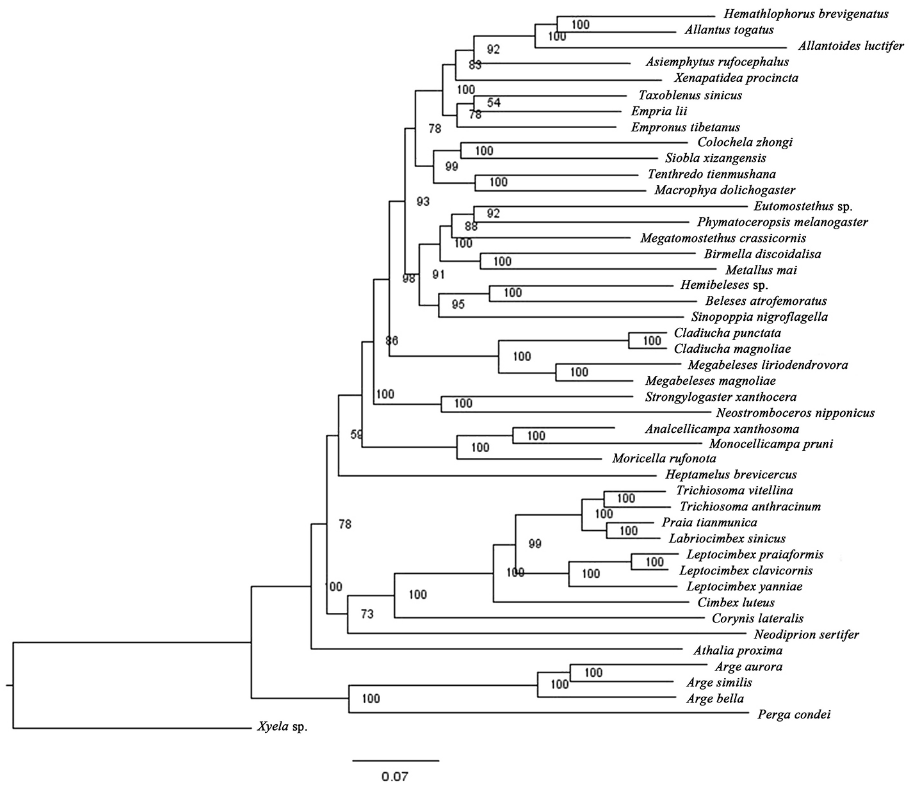

e

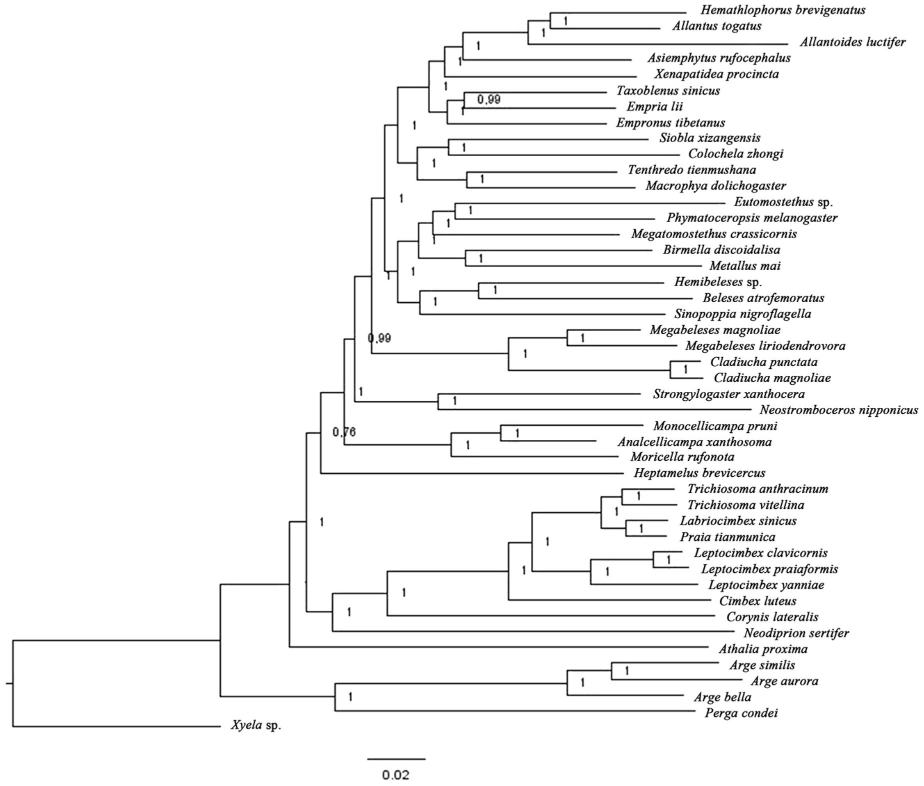

f

Figure S1 Phylogenetic tree recovered from the dataset of P12RNAexc3genes using **(a)** ML, **(b)** BI; of P123RNAexc3genes using **(c)** ML and (**d**) BI; of P12RNA using **(e)** ML and **(f)** BI. An optimal partitioning scheme was selected with PartitionFinder. The posterior probabilities and/or bootstrap values were shown in the trees

Table S1: Summary information of mitogenomes used in phylogenetic analyses.

	Species	Family	Subfamily	Gender	Locality	Accession number	References
Ingroup	*Allantoides luctifer*	Tenthredinidae	Allantinae	No information in NCBI	No information in NCBI	KJ713152	Wei *et al.,* 2016
	Allantus togatus			Female	SPAIN: Valencia: Parque Natural de la Sierra Calderona:Serra	MW464859	Yang *et al.,* 2021
	Asiemphytus rufocephalus			No information in NCBI	Songshan Forest Park, Beijing	KR703582	Song *et al.,* 2016
	Empria lii			Female	Dalongtan, Hubei	MW632124	This study
	Empronus tibetanus			Female	Songlinkou, Tibet Autonomous Region	MZ265343	This study
	Hemathlophorus brevigenatus			Female	Mount Wuyi, Fujian	MW632125	Liu J *et al.*, 2021
	Hemibeleses sp.			Female	Chanyuan Temple, Zhejiang	MZ265344	This study
	Taxoblenus sinicus			Female	MenYuan, Qinghai	MW632126	Sun *et al* ., 2021
	Xenapatidea procincta			No information in NCBI	Kaishan Old Temple, Zhejiang	MW487928	This study
	Beleses atrofemoratus		Blennocampinae	Female	Mount Qiyun, Hunan	MZ265347	This study
	Eutomostethus sp.			Female	Mount Lu, Jiangxi	MT663219	Li *et al.*, 2020
	Megatomostethus crassicornis			Female	Mount Tianmu, Zhejiang	MZ265345	This study
	Phymatoceropsis melanogaster			Male	Chanyuan Temple, Zhejiang	MZ265346	This study
	Birmella discoidalisa		Heterarthrinae	Female	Mount Emei, Sichuan	MF197548	Wu *et al.,* 2019
	Metallus mai			Female	Mount Lu, Jiangxi	MW255941	This study
	Sinopoppia nigroflagella			Female	Xinting Village, Zhejiang	MW487927	Wan *et al.,* 2021
	Colochela zhongi		Tenthrediniinae	Female	Mount Taibai, Shaanxi	MT702984	Wu *et al.,* 2020

Macrophya dolichogaster			Female	Jiulong Wetland, Zhejiang	MW544890	This study
Siobla xizangensis			Female	Motuo, Tibet Autonomous Region	MN562486	Luo *et al.,* 2019
Tenthredo tienmushana			No information in NCBI	No information in NCBI	KR703581	Song *et al.,* 2015
Cladiucha magnoliae		Megabelesesinae	Female	Sangzhi, Hunan	MT295305	This study
Cladiucha punctata			Female	Mount Qiyun, Hunan	MT295306	This study
Megabeleses liriodendrovorax			Unknown	Mount Dawei, Hunan	MW255939	This study
Megabeleses magnoliae			Male	Chanyuan Temple, Zhejiang	MW255940	This study
Neostromboceros nipponicus		Selandriinae	Female	Mount Dayi, Hunan	MW632127	This study
Strongylogaster xanthocera			Female	Bao Tianman Forest Meteorological Station, Henan	MW324676	Liu Y *et al.,* 2021
Analcellicampa xanthosoma		Nematinae	Female	Mount Yun, Wugang, Hunan	MH992752	Niu *et al.,* 2019
Monocellicampa pruni			Female	Shanghe, Shandong	JX566509	Wei *et al.,* 2015
Moricella rufonota			Female	Xinting village, Zhejiang	MW487926	Tan *et al.,* 2021
Athalia proxima		Athaliinae	Female	Dalbergia Forest Farm, Zhejiang	MN527306	He *et al.,* 2019
Cimbex luteus		Cimbicinae	Female	Nanguan District, Jilin	MW136447	Yan *et al.,* 2021
Labriocimbex sinicus			Male	Shaoyang, Hunan	MH136623	Yan *et al.,* 2019
Leptocimbex clavicornis			Female	Songlinkou, Tibet Autonomous Region	MT478109	Cheng et al., 2018
Leptocimbex praiaformis	Cimbicidae		Male	Guyuan, Ningxia	MT478110	Cheng et al., 2018
Leptocimbex yanniae			Female	Longcanggou, Sichuan	MT478111	Cheng et al., 2018
Praia tianmunica			Female	Mount Tianmu, Zhejiang	MT665975	Cheng *et al.,* 2020
Trichiosoma anthracinum			No information in NCBI	Hanmi, Tibet Autonomous Region	KT921411	Song *et al.,* 2016

	Trichiosoma vitellina			Female	Songjiang Town, Jilin	MN853777	Chen *et al.,* 2020
	Corynis lateralis		Corynidinae	No information in NCBI	No information in NCBI	KY063728	Doğan and Korkmaz, 2017
	Neodiprion sertifer	Diprionidae	Diprioninae	Larvae	Turkey: Manisa	MK994526	Aydemir and Korkmaz, 2019
	Heptamelus brevicercus	Heptamelidae		Female	Mount Yun, Wugang, Hunan	MW632128	This study
	Arge bella		Arginae	Female	Mount Qiyun, Hunan	MF287761	Du *et al.,* 2018
	Arge similis	Argidae		No information in NCBI	No information in NCBI	MG923484	Tang *et al.,* 2019
	Arge sp./Arge aurora			Female	Mount Lu, Jiangxi	MN913350	Wu *et al.,* unpublished
	Perga condei	Pergidae	Perginae	No information in NCBI	No information in NCBI	AY787816	Castro and Dowton, 2005
Outgroup	*Xyela sp*	Xyelidae		Male	Mounts Qin, Shaanxi	MG923517	Tang et al. 2018

Table S2: Best partitioning scheme and model selected by PartitionFinder for phylogenetic analyses.

Subset	Partition scheme	Model
**P1**	rrnS, nad4l 1st, nad4l 2nd, nad1 1st, nad5 1st, nad4 1st	GTR+I+G
**P2**	rrnL, atp8 2nd, atp8 1st, nad6 1st, nad2 1st, trn C, trn P, trn V, trn S1, trn E, trn H, nad3 1st, trn I, trn N, trn T, trn M, trn G, trn L1, trn W, trn A, trn Y, trn R, trn F, trn S2, trn L2, trn Q, trn D	GTR+I+G
**P3**	cytb 3rd, cox1 3rd, cox2 3rd, cox3 3rd,nad3 3rd, nad2 3rd, atp6 3rd, nad6 3rd, atp8 3rd	GTR+I+G
**P4**	nad3 2nd, cox1 1st, cox2 1st, trn K, cytb 1st, cox3 1st, atp6 1st	GTR+I+G
**P5**	nad5 2nd, nad1 2nd, nad4 2nd, nad2 2nd, nad6 2nd, cox2 2nd, cytb 2nd, cox1 2nd, atp6 2nd, cox3 2nd	GTR+I+G
**P6**	nad1 3rd, nad5 3rd, nad4L 3rd, nad4 3rd	HKY+I+G

The best partitioning scheme selected by PartitionFinder v1.1.1 for different dataset.

Table S3: Regression of the pairwise distances of the different genes and codon positions in the Megabelesesinae species.

Gene	Regression	R Average	GTR distance
atp6	y = 1.0296x - 0.0021	0.999	0.290
atp8	y = 3E-09x + 0.5079	0.010	10.087
cox1	y = 0.9925x + 0.0006	1.000	0.196
cox2	y = 0.9809x + 0.0008	1.000	0.240
cox3	y = 0.9877x + 0.0008	0.999	0.251
cytb	y = 0.9858x + 0.0009	0.999	0.254
nd1	y = 0.9527x + 0.005	1.000	0.242
nd2	y = 0.8733x + 0.0264	0.996	0.439
nd3	y = 0.9647x + 0.003	0.998	0.302
nd4	y = 0.9426x + 0.0067	0.999	0.274
nd4l	y = 2E-09x + 0.3119	0.177	6.156
nd5	y = 0.9363x + 0.0086	0.999	0.303
nd6	y = 0.2133x + 0.1773	0.376	4.490
rrnL	y = 0.786x + 0.043	0.994	0.237
rrnS	y = 0.8016x + 0.0409	0.992	0.243
tRNAs	y = 0.9612x + 0.0034	1.000	0.164
1st codon position	y = 0.9898x + 0.0008	1.000	0.266
2nd codon position	y = 0.9744x + 0.0016	1.000	0.135
1st and 2nd codon position	y = 0.9833x + 0.0016	1.000	0.197
3rd codon position	y = 0.7093x + 0.0589	0.979	0.587

Regression of the pairwise distances of the different genes and codon positions in the *Cladiucha* and *Megabeleses*

Table S4: Mitogenome organisations of Megabelesesinae species.

	Megabeleses liriodendrovorax								*Megabeleses magnoliae*						
**Gene**	**Strand**	**Start**	**Stop**	**Length (bp)**	**Start codon**	**Stop codon**	**IGN**	**Gene**	**Strand**	**Start**	**Stop**	**Length (bp)**	**Start codon**	**Stop codon**	**IGN**
trnM	J	1	69	69			5	*trnM*	J	1	69	69			7
trnQ	J	75	143	69			29	*trnQ*	J	77	145	69			18
trnI	J	173	238	66			8	*trnI*	J	164	229	66			22
nad2	J	247	1296	1050	ATG	TAA	2	*nad2*	J	252	1298	1047	ATG	TAA	2
trnW	J	1299	1367	69			-1	*trnW*	J	1301	1368	68			-8
trnC	N	1367	1432	66			1	*trnC*	N	1361	1428	68			4
trnY	N	1434	1500	67			10	*trnY*	N	1433	1501	69			9
cox1	J	1511	3046	1536	ATA	TAA	1	*cox1*	J	1511	3046	1536	ATA	TAA	8
trnL2	J	3048	3115	68			0	*trnL2*	J	3055	3122	68			0
cox2	J	3116	3796	681	ATA	TAA	9	*cox2*	J	3123	3797	675	ATT	TAA	4
trnK	J	3806	3876	71			0	*trnK*	J	3802	3872	71			2
trnD	J	3877	3941	65			0	*trnD*	J	3875	3941	67			0
atp8	J	3942	4106	165	ATT	TAA	-7	*atp8*	J	3942	4103	162	ATT	TAA	-7
atp6	J	4100	4777	678	ATG	TAA	-1	*atp6*	J	4097	4774	678	ATG	TAA	-1
cox3	J	4777	5565	789	ATG	TAA	12	*cox3*	J	4774	5559	786	ATG	TAG	3
trnG	J	5578	5640	63			0	*trnG*	J	5563	5628	66			0
nad3	J	5641	5997	357	ATT	TAA	3	*nad3*	J	5629	5985	357	ATT	TAA	1
trnA	J	6001	6067	67			1	*trnA*	J	5987	6055	69			36
trnN	J	6069	6137	69			0	*trnN*	J	6092	6160	69			0
trnS1	J	6138	6204	67			2	*trnS1*	J	6161	6227	67			2
trnE	J	6207	6273	67			14	*trnE*	J	6230	6295	66			286
trnR	N	6288	6354	67			2	*trnR*	N	6582	6649	68			2
trnF	N	6357	6424	68			6	*trnF*	N	6652	6719	68			3
nad5	N	6431	8149	1719	ATT	TAA	0	*nad5*	N	6723	8438	1716	ATT	TAA	0
trnH	N	8150	8214	65			3	*trnH*	N	8439	8502	64			0
nad4	N	8218	9564	1347	ATA	TAA	-4	*nad4*	N	8503	9847	1345	ATA	T	-4
nad4L	N	9561	9854	294	ATT	TAA	2	*nad4L*	N	9844	10137	294	ATT	TAA	2
trnT	J	9857	9920	64			0	*trnT*	J	10140	10204	65			0
trnP	N	9921	9986	66			1	*trnP*	N	10205	10269	65			1

nad6	J	9988	10506	519	ATT	TAA	-1	*nad6*	J	10271	10789	519	ATT	TAA	-1
cob	J	10506	11640	1135	ATG	T	0	*cob*	J	10789	11923	1135	ATG	T	0
trnS2	J	11641	11709	69			12	*trnS2*	J	11924	11992	69			49
nad1	N	11722	12672	951	ATT	TAA	0	*nad1*	N	12042	12992	951	ATT	TAA	0
trnL1	N	12673	12741	69			0	*trnL1*	N	12993	13060	68			0
rrnL	N	12742	14086	1345			0	*rrnL*	N	13061	14423	1363			0
trnV	N	14087	14146	60			0	*trnV*	N	14424	14484	61			0
rrnS	N	14147	15155	1009			0	*rrnS*	N	14485	15450	966			0
D-loop	N	15156	15466	311				D-loop		15451	16219	769			

Cladiucha magnoliae								*Cladiucha punctata*							
**Gene**	**Strand**	**Start**	**Stop**	**Length (bp)**	**Start codon**	**Stop codon**	**IGN**	**Gene**	**Strand**	**Start**	**Stop**	**Length (bp)**	**Start codon**	**Stop codon**	**IGN**
trnM	J	1	69	69			9	*trnM*	J	1	69	69			9
trnQ	J	79	147	69			8	*trnQ*	J	79	147	69			22
trnI	J	156	222	67			25	*trnI*	J	170	236	67			50
nad2	J	248	1300	1053	ATG	TAA	5	*nad2*	J	287	1336	1050	ATG	TAA	14
trnW	J	1306	1372	67			-1	*trnW*	J	1351	1417	67			0
trnC	N	1372	1440	69			30	*trnC*	N	1418	1486	69			42
trnY	N	1471	1535	65			8	*trnY*	N	1529	1594	66			6
cox1	J	1544	3082	1539	ATT	TAA	26	*cox1*	J	1601	3136	1536	ATT	TAA	45
trnL2	J	3109	3177	69			0	*trnL2*	J	3182	3250	69			0
cox2	J	3178	3858	681	ATT	TAA	4	*cox2*	J	3251	3931	681	ATT	TAA	2
trnK	J	3863	3932	70			0	*trnK*	J	3934	4003	70			0
trnD	J	3933	3996	64			0	*trnD*	J	4004	4067	64			0
atp8	J	3997	4158	162	ATT	TAA	-7	*atp8*	J	4068	4229	162	ATT	TAA	-7
atp6	J	4152	4829	678	ATG	TAA	-1	*atp6*	J	4223	4900	678	ATG	TAA	-1
cox3	J	4829	5617	789	ATG	TAA	5	*cox3*	J	4900	5685	786	ATG	TAA	5
trnG	J	5623	5687	65			0	*trnG*	J	5691	5755	65			0
nad3	J	5688	6041	354	ATA	TAA	8	*nad3*	J	5756	6109	354	ATA	TAA	17
trnA	J	6050	6115	66			39	*trnA*	J	6127	6195	69			39
trnN	J	6155	6222	68			0	*trnN*	J	6235	6303	69			0
trnS1	J	6223	6289	67			7	*trnS1*	J	6304	6370	67			7

trnE	J	6297	6361	65			-1	*trnE*	J	6378	6442	65			-2
trnR	N	6361	6429	69			8	*trnR*	N	6441	6508	68			7
trnF	N	6438	6504	67			30	*trnF*	N	6516	6583	68			22
nad5	N	6535	8253	1719	ATT	TAA	0	*nad5*	N	6606	8324	1719	ATT	TAA	0
trnH	N	8254	8320	67			0	*trnH*	N	8325	8392	68			0
nad4	N	8321	9665	1345	ATA	T	-4	*nad4*	N	8393	9737	1345	ATA	T	-4
nad4L	N	9662	9955	294	ATT	TAA	2	*nad4L*	N	9734	10027	294	ATT	TAA	2
trnT	J	9958	10028	71			0	*trnT*	J	10030	10097	68			0
trnP	N	10029	10095	67			1	*trnP*	N	10098	10164	67			1
nad6	J	10097	10609	513	ATA	TAA	-1	*nad6*	J	10166	10678	513	ATA	TAA	-1
cob	J	10609	11742	1134	ATG	TAA	0	*cob*	J	10678	11811	1134	ATG	TAA	0
trnS2	J	11743	11810	68			38	*trnS2*	J	11812	11879	68			34
nad1	N	11849	12802	954	ATA	TAA	0	*nad1*	N	11914	12867	954	ATA	TAA	0
trnL1	N	12803	12870	68			0	*trnL1*	N	12868	12935	68			0
rrnL	N	12871	14214	1344			0	*rrnL*	N	12936	14276	1341			0
trnV	N	14215	14280	66			0	*trnV*	N	14277	14343	67			0
rrnS	N	14281	15223	943			0	*rrnS*	N	14344	15329	986			0
D-loop	N	15224	15761	538				D-loop	N	15330	16187	858			

Notes.
J and N refer to heavy and light strands, respectively; IGN refers to intergenic nucleotides. Minus indicates overlapping sequences between adjacent genes.

Table S5: Nucleotide composition of the mitogenomes of Megabelesesinae species.

Feature	*Species*	T%	C%	A%	G%	A+T%	G+C%	AT-skew	GC-skew
Whole	*C. punctata*	41.10	10.00	42.00	6.90	83.10	16.90	0.011	-0.183
genome	*C. magnoliae*	41.00	10.00	42.10	7.00	83.10	17.00	0.013	-0.176
	M. liriodendrovorax	38.90	11.70	41.80	7.60	80.70	19.30	0.036	-0.212
	M. magnoliae	39.70	10.90	42.10	7.30	81.80	18.20	0.029	-0.198
PCGs	*C. punctata*	45.30	9.20	36.30	9.10	81.60	18.30	-0.110	-0.005
	C. magnoliae	45.30	9.10	36.50	9.10	81.80	18.20	-0.108	0.000
	M. liriodendrovorax	44.50	10.20	34.90	10.40	79.40	20.60	-0.121	0.010
	M. magnoliae	45.00	9.50	35.40	10.00	80.40	19.50	-0.119	0.026
First	*C. punctata*	38.40	9.30	38.80	13.50	77.20	22.80	0.005	0.184
codon	*C. magnoliae*	38.80	9.20	38.40	13.60	77.20	22.80	-0.005	0.193
position	*M. liriodendrovorax*	38.10	9.70	37.40	14.80	75.50	24.50	-0.009	0.208
	M. magnoliae	38.50	9.50	37.50	14.50	76.00	24.00	-0.013	0.208
Second	*C. punctata*	50.30	15.70	21.60	12.40	71.90	28.10	-0.399	-0.117
codon	*C. magnoliae*	50.50	15.60	21.60	12.40	72.10	28.00	-0.401	-0.114
position	*M. liriodendrovorax*	49.10	16.20	21.70	13.00	70.80	29.20	-0.387	-0.110
	M. magnoliae	49.60	16.10	21.60	12.70	71.20	28.80	-0.393	-0.118
Third	*C. punctata*	47.20	2.60	48.60	1.60	95.80	4.20	0.015	-0.238
codon	*C. magnoliae*	46.60	2.40	49.70	1.40	96.30	3.80	0.032	-0.263
position	*M. liriodendrovorax*	46.50	4.80	45.50	3.20	92.00	8.00	-0.011	-0.200
	M. magnoliae	47.00	2.90	47.20	2.80	94.20	5.70	0.002	-0.018
PCGs-J	*C. punctata*	43.40	11.00	37.20	8.40	80.60	19.40	-0.077	-0.134
	C. magnoliae	43.40	10.90	37.40	8.40	80.80	19.30	-0.074	-0.130
	M. liriodendrovorax	41.70	12.50	36.50	9.20	78.20	21.70	-0.066	-0.152
	M. magnoliae	42.80	11.50	36.90	8.80	79.70	20.30	-0.074	-0.133
First	*C. punctata*	35.20	11.10	40.50	13.20	75.70	24.30	0.070	0.086
codon	*C. magnoliae*	35.50	11.20	40.10	13.20	75.60	24.40	0.061	0.082
position	*M. liriodendrovorax*	34.30	11.70	39.70	14.30	74.00	26.00	0.073	0.100
	M. magnoliae	40.30	9.60	40.50	9.60	80.80	19.20	0.002	0.000
Second	*C. punctata*	48.00	18.20	22.50	11.30	70.50	29.50	-0.362	-0.234
codon	*C. magnoliae*	48.20	17.90	22.60	11.40	70.80	29.30	-0.362	-0.222
position	*M. liriodendrovorax*	46.90	18.50	22.40	12.30	69.30	30.80	-0.354	-0.201
	M. magnoliae	41.20	15.00	31.90	11.90	73.10	26.90	-0.127	-0.115
Third	*C. punctata*	46.90	3.70	48.60	0.70	95.50	4.40	0.018	-0.682
codon	*C. magnoliae*	46.40	3.60	49.50	0.50	95.90	4.10	0.032	-0.756
position	*M. liriodendrovorax*	44.00	7.50	47.50	1.00	91.50	8.50	0.038	-0.765
	M. magnoliae	46.90	9.80	38.40	4.90	85.30	14.70	-0.100	-0.333
PCGs-N	*C. punctata*	48.40	6.30	34.90	10.30	83.30	16.60	-0.162	0.241
	C. magnoliae	48.40	6.10	35.20	10.30	83.60	16.40	-0.158	0.256
	M. liriodendrovorax	49.10	6.50	32.20	12.20	81.30	18.70	-0.208	0.305
	M. magnoliae	48.70	6.40	32.80	12.10	81.50	18.50	-0.195	0.308
First	*C. punctata*	43.60	6.30	36.10	13.90	79.70	20.20	-0.094	0.376
codon	*C. magnoliae*	44.10	6.10	35.70	14.20	79.80	20.30	-0.105	0.399

position	*M. liriodendrovorax*	44.20	6.50	33.60	15.70	77.80	22.20	-0.136	0.414
	M. magnoliae	47.00	2.30	41.90	8.80	88.90	11.10	-0.057	0.586
Second	*C. punctata*	54.00	11.90	20.10	14.10	74.10	26.00	-0.457	0.085
codon	*C. magnoliae*	54.30	11.90	19.90	13.90	74.20	25.80	-0.464	0.078
position	*M. liriodendrovorax*	52.50	12.60	20.70	14.20	73.20	26.80	-0.434	0.060
	M. magnoliae	45.90	8.00	31.00	15.10	76.90	23.10	-0.194	0.307
Third	*C. punctata*	47.70	0.80	48.60	2.90	96.30	3.70	0.009	0.568
codon	*C. magnoliae*	46.90	0.50	49.90	2.70	96.80	3.20	0.031	0.688
position	*M. liriodendrovorax*	50.50	0.60	42.30	6.70	92.80	7.30	-0.088	0.836
	M. magnoliae	53.10	9.00	25.50	12.3	78.60	21.30	-0.351	0.155
tRNA	*C. punctata*	41.40	6.80	43.00	8.70	84.40	15.50	0.019	0.123
genes	*C. magnoliae*	41.30	6.90	43.10	8.80	84.40	15.70	0.021	0.121
	M. liriodendrovorax	40.30	7.10	42.80	9.80	83.10	16.90	0.030	0.160
	M. magnoliae	40.40	7.00	42.90	9.70	83.30	16.70	0.030	0.162
rRNA	*C. punctata*	43.60	4.80	41.90	9.70	85.50	14.50	-0.020	0.338
genes	*C. magnoliae*	43.50	5.10	41.80	9.60	85.30	14.70	-0.020	0.306
	M. liriodendrovorax	43.50	5.20	40.20	11.00	83.70	16.20	-0.039	0.358
	M. magnoliae	44.50	5.10	40.20	10.20	84.70	15.30	-0.051	0.333

Table S6: Relative synonymous codon usage (RSCU) of the Megabelesesinae mitogenomes.

	M. magnoliae	*M. liriodendrovorax*	*C. punctata*	*C. magnoliae*
UUU(F)	1.88	1.84	1.91	1.88
UUC(F)	0.12	0.16	0.09	0.12
UUA(L)	5.14	5.01	5.26	5.23
UUG(L)	0.28	0.30	0.15	0.18
CUU(L)	0.25	0.35	0.24	0.29
CUC(L)	0.00	0.00	0.01	0.00
CUA(L)	0.33	0.35	0.34	0.30
CUG(L)	0.01	0.00	0.00	0.00
AUU(I)	1.93	1.86	1.93	1.96
AUC(I)	0.07	0.14	0.07	0.04
AUA(M)	1.85	1.87	1.91	1.92
AUG(M)	0.15	0.13	0.09	0.08
GUU(V)	1.51	1.61	1.74	1.64
GUC(V)	0.03	0.12	0.00	0.04
GUA(V)	2.36	2.12	2.11	2.29
GUG(V)	0.10	0.15	0.15	0.04
UCU(S)	2.36	2.38	2.26	2.19
UCC(S)	0.21	0.25	0.14	0.25
UCA(S)	2.76	2.36	2.77	2.72
UCG(S)	0.02	0.02	0.02	0.00
CCU(P)	2.34	2.49	2.44	2.30
CCC(P)	0.20	0.39	0.23	0.17
CCA(P)	1.40	1.11	1.27	1.50
CCG(P)	0.07	0.00	0.07	0.03
ACU(T)	2.00	1.78	1.65	1.60
ACC(T)	0.08	0.33	0.09	0.17
ACA(T)	1.86	1.86	2.24	2.23
ACG(T)	0.06	0.03	0.03	0.00
GCU(A)	2.23	2.11	1.85	1.88
GCC(A)	0.12	0.48	0.25	0.21
GCA(A)	1.62	1.33	1.85	1.92
GCG(A)	0.04	0.07	0.04	0.00
UAU(Y)	1.88	1.77	1.87	1.89
UAC(Y)	0.12	0.23	0.13	0.11
UAA(*)	0.00	0.00	0.00	0.00
UAG(*)	0.00	0.00	0.00	0.00
CAU(H)	1.85	1.72	1.97	1.86
CAC(H)	0.15	0.28	0.03	0.14
CAA(Q)	1.83	1.93	1.87	1.97
CAG(Q)	0.17	0.07	0.13	0.03
AAU(N)	1.81	1.83	1.89	1.90
AAC(N)	0.19	0.17	0.11	0.10

AAA(K)	1.82	1.74	1.94	1.93
AAG(K)	0.18	0.26	0.06	0.07
GAU(D)	1.82	1.60	1.86	1.96
GAC(D)	0.18	0.40	0.14	0.04
GAA(E)	1.95	1.84	1.94	1.92
GAG(E)	0.05	0.16	0.06	0.08
UGU(C)	2.00	1.89	1.83	1.92
UGC(C)	0.00	0.11	0.17	0.08
UGA(W)	1.94	1.83	1.89	2.00
UGG(W)	0.06	0.17	0.02	0.00
CGU(R)	1.17	1.25	0.85	1.02
CGC(R)	0.00	0.00	0.09	0.00
CGA(R)	2.58	2.58	3.06	2.98
CGG(R)	0.25	0.17	0.00	0.00
AGU(S)	0.51	0.74	0.37	0.37
AGC(S)	0.07	0.07	0.05	0.00
AGA(S)	1.99	2.06	2.38	2.37
AGG(S)	0.07	0.12	0.02	0.09
GGU(G)	0.77	0.72	0.79	0.85
GGC(G)	0.04	0.02	0.09	0.02
GGA(G)	2.81	2.77	2.93	3.03
GGG(G)	0.37	0.49	0.19	0.09
